# Genome-Wide Analysis of Auxin Response Factors in Lettuce (*Lactuca sativa* L.) Reveals the Positive Roles of LsARF8a in Thermally Induced Bolting

**DOI:** 10.3390/ijms232113509

**Published:** 2022-11-04

**Authors:** Manman Hu, Zhengyang Qi, Zheng Ren, Jing Tong, Baoju Wang, Zhanhui Wu, Jinghong Hao, Ning Liu

**Affiliations:** 1 National Engineering Research Center for Vegetables, Key Laboratory of Urban Agriculture (North China), Institute of Vegetable Sciences, Beijing Academy of Agriculture and Forestry Sciences, Beijing 100097, China; 2 Beijing Key Laboratory of New Technology in Agricultural Application, National Demonstration Center for Experimental Plant Production Education, Plant Science and Technology College, Beijing University of Agriculture, Beijing 102206, China

**Keywords:** auxin response factor, *Lactuca sativa*, genome-wide analysis, ARF8, thermally induced bolting, gene expression, thermal stress

## Abstract

Warm temperatures induce plant bolting accompanied by flower initiation, where endogenous auxin is dynamically associated with accelerated growth. Auxin signaling is primarily regulated by a family of plant-specific transcription factors, AUXIN RESPONSE FACTORS (ARFs), which either activate or repress the expression of downstream genes in response to developmental and environmental cues. However, the relationship between ARFs and bolting has not been completely understood in lettuce yet. Here, we identified 24 *LsARF*s (*Lactuca sativa* ARFs) in the lettuce genome. The phylogenetic tree indicated that LsARFs could be classified into three clusters, which was well supported by the analysis of exon–intron structure, consensus motifs, and domain compositions. RNA-Seq analysis revealed that more than half of the *LsARF*s were ubiquitously expressed in all tissues examined, whereas a small number of LsARFs responded to UV or cadmium stresses. qRT-PCR analysis indicated that the expression of most *LsARF*s could be activated by more than one phytohormone, underling their key roles as integrative hubs of different phytohormone signaling pathways. Importantly, the majority of *LsARF*s displayed altered expression profiles under warm temperatures, implying that their functions were tightly associated with thermally accelerated bolting in lettuce. Importantly, we demonstrated that silencing of *LsARF8a,* expression of which was significantly increased by elevated temperatures, resulted in delayed bolting under warm temperatures, suggesting that *LsARF8a* might conduce to the thermally induced bolting. Together, our results provide molecular insights into the *LsARF* gene family in lettuce, which will facilitate the genetic improvement of the lettuce in an era of global warming.

## 1. Introduction

The phytohormone auxin (indole-3-acetic acid) is involved in a multitude of growth and developmental processes in land plants. In the past decade, auxin perception and signaling pathways that mediate the changes in gene expression have been explored intensively, leading to the identification of three dedicated protein families [1,2,3]. Theoretically, in the absence of auxin, the pathway is repressed by a group of AUXIN/INDOLE-3-ACETIC ACID (Aux/IAA) inhibitors. Aux/IAA proteins physically bind to the AUXIN RESPONSE FACTORs (ARFs) that allow the expression of auxin-dependent genes. To unlock the pathway, Aux/IAA proteins, in the presence of auxin, were targeted to the F-box-containing TRANSPORT INHIBITOR RESPONSE and AUXIN-SIGNALING F-BOX PROTEIN (TIR1/AFB) ubiquitin ligases, resulting in their degradation by the 26S proteasome [4]. Subsequently, de-repression of ARF transcription factors allows them to directly mediate the transcription of their downstream genes [5]. As ARF proteins couple the perception of the hormone auxin to gene expression programs, this represents the core of the auxin signaling pathway.

It is well established that ARFs play crucial roles in almost every developmental aspect such as flowering and bolting in Arabidopsis [6]. For instance, AtARF1 and AtARF2 were involved in the regulation of floral organ abscission, while AtARF3/ETTIN functions in gynoecium morphogenesis, self-incompatibility, and flower development [7,8,9]. Genetic analysis suggested that AtARF6 and AtARF8 redundantly regulate the flower maturation in Arabidopsis as well as tomato [10,11,12]. In addition to their developmental roles, recent studies found that some ARFs were involved in plant responses to abiotic stress. Overexpression of *AtARF3* stimulated the expression of a set of drought-stress-responsive genes including *RAP2.6*, *NAC019*, *ERD5*, *ZAT12,* and *RD26* in Arabidopsis; conversely, these ARF-activated genes remained at very lower expression levels in *arf3* mutants in response to the drought stress [13]. Similarly, expression levels of the majority of *SlARF*s were attenuated under salinity stress, whereas several *SlARF* genes such as *SlARF1*, *SlARF4*, and *SlARF19* exhibited significant upregulation upon salt stress [14]. Thus, these observations highlighted the neglected roles of ARFs that might also participate in the plant response to abiotic stress [15]. In our previous studies, we showed that the transcript accumulation of *LsARF2* and *LsARF5* in flower stalk kept increasing with the progression of lettuce bolting and revealed that tryptophan metabolism pathways were involved in the early bolting under high temperatures in lettuce [16,17]. These observations are consistent with the fact that the auxin content in stem tissues increased when flower stalks initiated their elongation [17]. As the major components of auxin signaling, LsARFs might integrate thermal and floral signals to coordinate floral development and plant growth in lettuce. Given that ARFs are tightly associated with multiple developmental and environmental cues, it is reasonable that they might be the potential intersections between two signaling pathways, wherein plant optimizes their developmental strategy to tackle the thermal challenges.

Lettuce (*Lactuca sativa* L.) is a globally important leaf vegetable in the family Asteraceae (Compositae) cultivated preliminarily for its fleshy leaves or succulent stems [18]. Lettuce growth is sensitive to heat stress, and exposure of lettuce to warm temperatures can reduce vegetative growth by hastening floral transition (premature bolting and accelerated flowering), ultimately resulting in severe economic loss to world producers, especially during summer productions [19,20]. Although ARFs probably link thermal response and floral transition signaling, the involvement of ARFs in lettuce flowering induction by warm temperatures remains largely unknown thus far.

As of 2020, the world production of lettuce (chicory included) reached 27.7 million tons, and China is ranked the highest in lettuce production, followed by USA and India [21]. Despite the economic importance of lettuce, a genome-wide, systematic study of the lettuce *ARF* gene family has not been reported yet. The first annotated genome of lettuce was released in 2017, and afterward, genome sequencing of 445 lettuce varieties showed the origins and breeding history of the crop [22,23]. The genome information facilitates the research and enables us to conduct genome-wide analysis of ARFs in lettuce. In the present study, a genome-wide, comprehensive analysis of lettuce ARF genes was performed, and a total of 24 *LsARF* genes were identified. The physical and chemical characteristics, genomic structures, chromosomal locations, evolutionary relationships, and expression profiles of the *LsARF* gene family were investigated in detail. Finally, the virus-induced gene silencing (VIGS) approach was employed to analyze the biological roles of *LsARF8a* under thermally induced bolting. These findings provide deep insights into the function of *LsARF* genes during the lettuce bolting upon warm temperatures.

## 2. Results

### 2.1. Identification of LsARFs from the Lettuce Genome

To identify lettuce *ARF* genes, protein sequences of functionally validated *ARF*s from Arabidopsis and rice were used as the queries to perform BLASTp searches against the lettuce genome. After removing the non-representative splicing forms of the same gene locus, 27 *ARF*-like genes were obtained from the genome sequences of *L. sativa*. Further, the presence of the B3 DNA-binding and AuxRE domains in these ARF-like proteins were scanned using the conserved domain search (CD-search) with an e-value <10^−10^. Due to the lack of an AuxRE motif, three genes (Lsat_1_v5_gn_5_62560, Lsat_1_v5_gn_6_46381, and Lsat_1_v5_gn_8_118440) were removed from our analysis, and ultimately, a total of 24 genes were identified as *LsARF*s. According to the homologies against Arabidopsis ARFs (Figure S1), the nomenclature of these LsARF genes are listed in Table 1. The predicted proteins encoded by *LsARF*s varied from 432 amino acids (LsARF3b) to 1025 amino acids (LsARF7a), with corresponding molecular weights from 47.53 to 115.76 kDa. Of these putative LsARF proteins, the theoretical isoelectric points ranged from 5.32 (LsARF5b) to 7.93 (LsARF16d), indicating that they could participate in biochemical processes under distinct in vivo environments.

### 2.2. Chromosomal Distribution and Duplication Events among LsARF Genes

**Figure 1:**
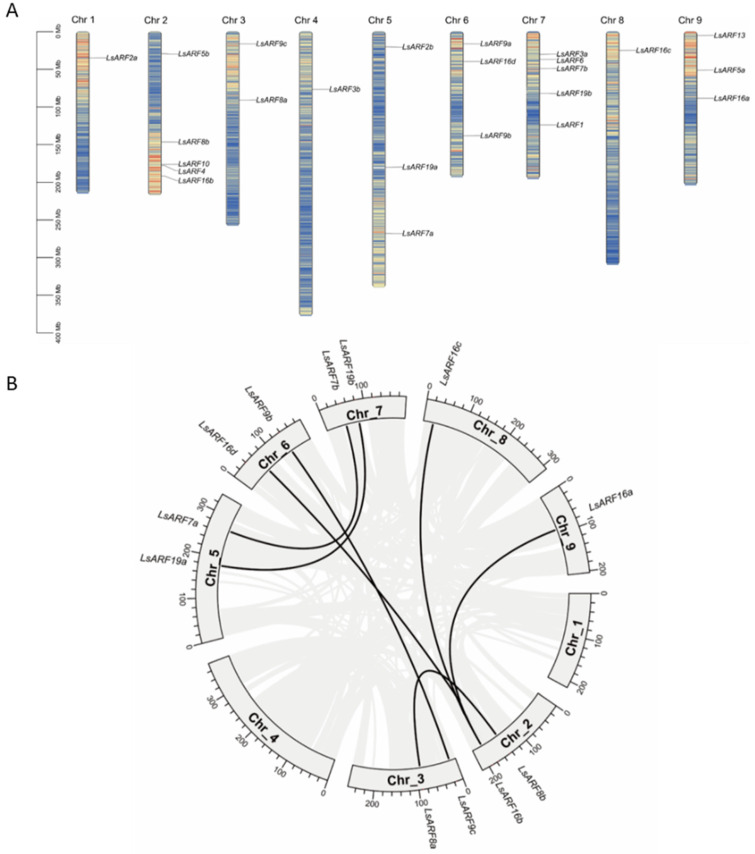
Chromosomal distribution and duplication events of *LsARF* genes. (**A**) Chromosomal location of members of the *LsARF* gene family. The chromosome numbers and size are indicated at the top and bottom of each bar, respectively. The number on the right side of the bars designated the approximate physical position of the first exon of corresponding *LsARF* genes on lettuce chromosomes. (**B**) Mapping of duplicated *LsARF* genes on the lettuce genome. Grey ribbons indicate a collinear relationship among the blocks in the whole genome, and black ribbons show *LsARF* paralogs.

The physical map position of *LsARF* genes on nine lettuce chromosomes was established. Chromosomes 2 and 7 contain the largest number of *LsARF*s, comprising six members; chromosome 5, 6, and 9 each contain two members; chromosome 6 has one member; and chromosome 1, 4, and 8 each contain a single *LsARF*. The number of *LsARF*s in the lettuce genome is similar to their counterparts in Arabidopsis. Pairwise sequence comparison of *LsARF* proteins suggests that the homology broadly ranged from 21.40% (*LsARF3a* and *LsARF7b*) to 79.52% (*LsARF2a* and *LsARF2b*). Strikingly, *LsARF* members in the *ARF9* subclade comprising LsARF9a/9b/9c share high sequence identity (76.35–79.04%), suggesting that these LsARFs are likely to be originated from gene duplications while they are positioned to different chromosomes. A similar event was found in *ARF10/16* subclade, which includes *LsARF16a/16b/16c/16d/10* with identity from 57.72% to 70.85%. Duplication analysis revealed the expansion of the ARF gene family in lettuce (Figure 1B). Among 24 *LsARF*s, 10 were found to be segmentally duplicated, such as *LsARF7a/7*b, *LsARF8a/8b*, *LsARF16a/16b/16/c/16d*, and *LsARF19a/19b*. Accordingly, high sequence similarity between duplicated gene pairs was observed, suggesting they might participate in the regulation of similar biological processes. Interestingly, tandem duplication did not occur in *LsARF* genes, whereas it was common in other plant species such as *AtARF*s.

### 2.3. Structural Analysis and Conserved Motifs of LsARF Genes

**Figure 2:**
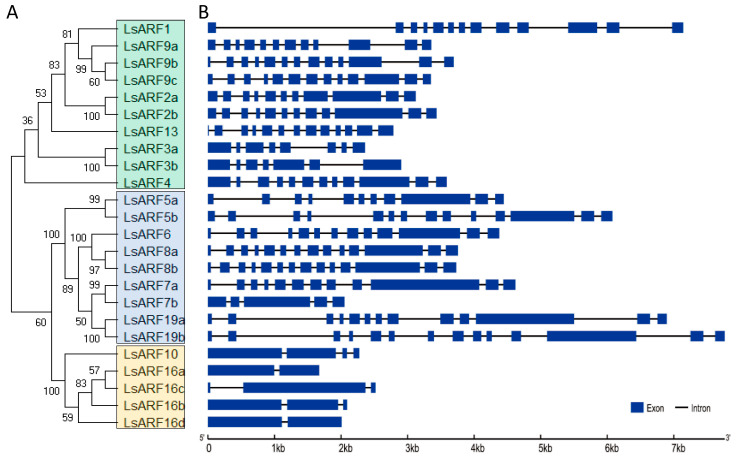
Classification of *L. sativa* ARF proteins. (**A**). Neighbor-joining tree were generated using MEGA to determine the phylogenetic relationship between *L. sativa* ARF proteins (left). According to classification proposed by Finet et al. (2013), LsARFs were divided into three subgroups: A (top), B (middle) and C (bottom), and shadow colors were used to distinguish different LsARF subgroups. (**B**). The intron–exon organization of *LsARF* genes was plotted using Gene Structure Display Server (Version 2.0). Blue boxes represent exons, and grey lines represent introns.

To better understand the structure of the exon and intron, the full-length cDNA sequences of *LsARF* genes were aligned against the corresponding genome DNA sequences (Figure 2). Gene structure analysis suggested that the number of exons of *LsARF*s ranged from 2 to 14. It is noteworthy that *LsARF* members in the *ARF8* and *ARF9* subfamily share identical intron–exon structure, suggesting they are highly conserved within the same subclade. Three members of the *LsARF10/16* subclade also exhibited similar gene structures, despite the fact that *LsARF16a* is a truncated gene. Although the exon–intron structure of *LsARF*s varies between subclades, it is similar within subclades, which was supported by the phylogenetic tree constructed with the cDNA sequences of *LsARF*s.

**Figure 3:**
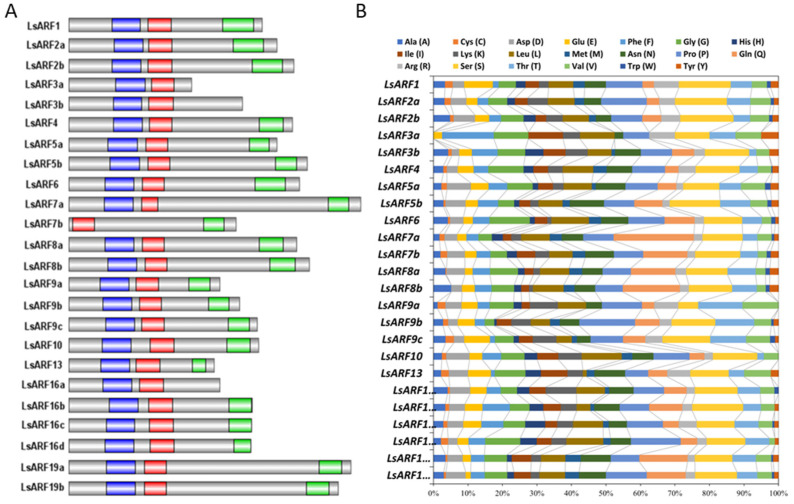
Analysis of conserved domains in LsARF porteins. (**A**) Schematic organization of conserved domains in LsARF proteins. The B3 DNA-binding domain, Aux_Resp domain, and PB1 domain are shown in blue, red, and green, respectively. (**B**) Amino acid composition of MR domains in LsARF proteins. Bars represent the percentage of different amino acid residues in MR domains of LsARFs, and each color represents one kind of amino acid.

Furthermore, we performed multiple sequence alignment using the deduced amino acid sequences of *LsARF* genes. As shown in Figure 3A, most LsARF proteins contain highly conserved DBD domains at N-terminal and PB1 domain at C-terminal. LsARF7b had a truncated DBD domain in which the B3 region was deleted, indicating that its DNA-binding ability could be impaired despite the remaining AuxRE motif. The PB1 domain of LsARF3a, LsARF3b, and LsARF16a was completely lost, suggesting that it might be independent of IAA/AUX-induced degradation. Although the DBD and PB1 domains are highly conserved, the MRs showed the highest divergence in the amino acid composition of ARF proteins. On the basis of the enrichment of specific amino acids in the MR, as well as on the ability of some tested AtARFs in transient experiments, it is predictable whether ARFs act as activators or repressors. Composition analysis of LsARF proteins suggest that the MRs of LsARF7a/7b, LsARF6, and LsARF19a/19b are abundant in Q residues (Figure 3B), thereby suggesting they might be transcriptional activators.

To identify the novel conserved motif, the MEME online program was used to analyze the motif distribution of LsARF proteins (Figure S2). In our analysis, eight motifs were identified from LsARF proteins. Among them, motifs 1, 2, 3, and 6 correspond to the B3 domain (PF02362); motifs 5, 7, and 8 correspond to the Auxin_Resp (Pf06507); and motif 4 represents the PB1 domain (PF00564). Motifs 3 and 5 are conserved among LsARFs, suggesting that they might be novel motifs essential to ARF proteins. Furthermore, LsARFs in the same subfamily shared similar patterns of motif composition, indicating their functional similarities. Thus, the distribution of the motifs also revealed that LsARFs were likely conserved during evolution.

### 2.4. Analysis of Cis-Elements in the Promoter Regions and Gene Ontology

**Figure 4:**
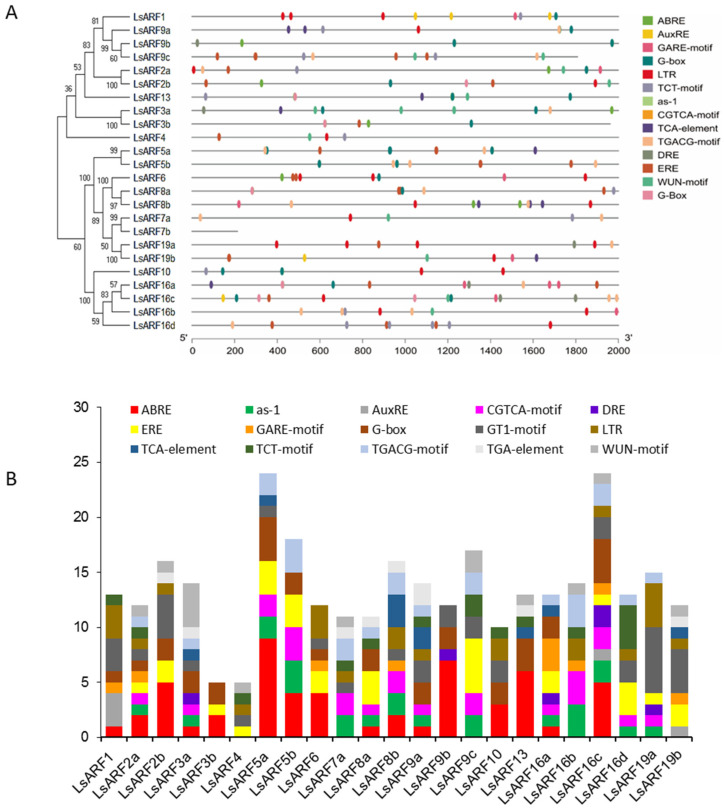
Identification of the cis-acting elements in the putative promoter region of *LsARF* genes. (**A**) The distribution of 15 major cis-elements in the putative promoter of *LsARF* genes. Fifteen cis-elements including auxin-responsive elements (AuxRE), as-1, ABA-responsive element (ABRE), CGTGCA motif, dehydration-responsive element (DRE), ethylene-responsive element (ERE), gibberellin-responsive motif (GARE), G-box, GT1-motif, low-temperature-responsive element (LRE), TCA-element, TCT-motif, TGACG motif, TGA element, and wound-responsive element (WUN) are designated with different colors. (**B**) Number of different cis-elements in the *LsARF* promoter regions.

In order to identify the potential regulatory regime, we analyzed the cis-elements in the 2 kb upstream of all identified *LsARF* genes by using the PlantCare database (Figure 4A). The promoter regions of *LsARF3b*, *LsARF7b*, and *LsARF9c* fall in the sequencing gap that contains artificially filled ambiguous N nucleotides, and thus removal of these regions resulted in reduced size of these promoter sequences. Our analysis identified 305 major cis-acting elements in these putative *LsARF* promoter regions (Figure 4B), and the five most highly represented cis-elements were the ABA-responsive element (ABRE, 54), GT1-motif (GT1, 36), G-box (32), ethylene-responsive element (ERE, 30), and low-temperature-responsive element (LTR, 24). As the promoter sequence of *LsARF7b* is very short, no motif was identified in these regions, and *LsARF7b* was excluded from the analysis. Surprisingly, ABRE was found in 17 out of 23 LsARF members, and 11 of them contain more than one such element. The auxin-responsive elements (AuxRE) were only identified in three *LsARF* members, while TGACG-motif, found to be associated with auxin response, was presented in the promoter of many *LsARF* genes. Gibberellin-responsive element (GARE) was found in eight members of the LsARF gene family. A total of 46 elements involved in MeJA-responsiveness (CGTAC and TGACG) were found in the promoter regions of almost all LsARF members, and similarly, 33 cis-elements (*as-1* and TCA motif) were identified as being involved in the salicylic-acid-responsiveness elements. The results reveal that these lettuce ARFs might be involved in the hormonal crosstalk due to the presence of such hormone-responsive motifs.

### 2.5. Phylogenetic Analysis of LsARFs

**Figure 5:**
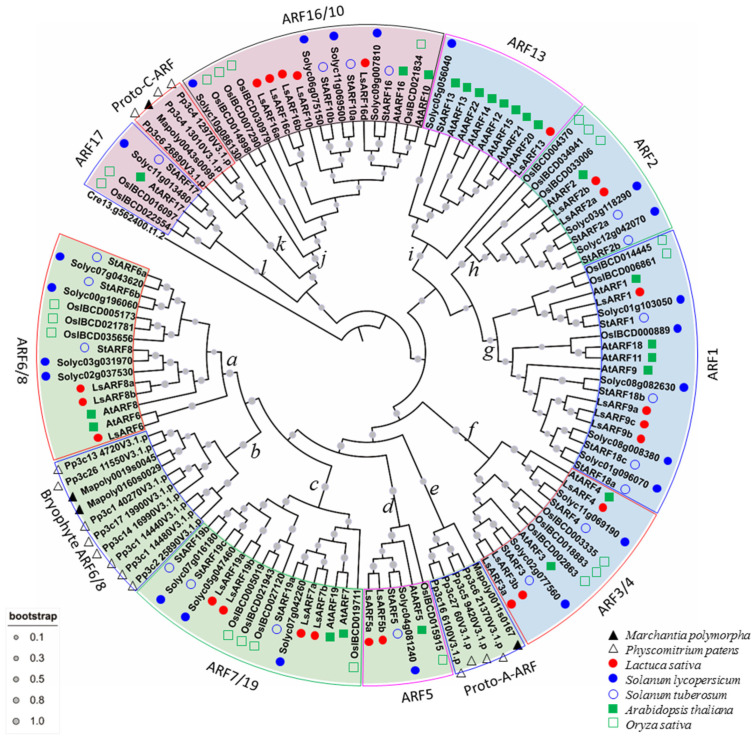
Phylogenetic relationship among ARF proteins from liverwort, moss, tomato, potato, Arabidopsis, rice, and lettuce. The deduced full-length amino acid sequences of ARFs from liverwort, moss, tomato, potato, Arabidopsis, rice, and lettuce were aligned by the MUSCLE method, and the neighbor-joining tree was generated using MEGA X to determine the phylogenetic relationship between those ARF proteins. According to the classification proposed by Mutte et al. (2017), LsARFs were divided into three subgroups: A (purple), B (blue), and C (green). Shadow colors are used to distinguish different LsARF subgroups.

On the basis of ARF protein sequences from liverwort (*Marchantia polymorpha*), moss (*Physcomitrella patens*), tomato, potato, rice, and Arabidopsis, a phylogenetic tree was constructed to explore the evolutionary scenario of ARF genes (Figure 5). One B3 domain-containing sequence (Cre13g562400) from green algae (*Chlamydomonas reinhardtii*) was used as an outgroup. Consistent with analyses by Mutte et al. (2018), 131 ARFs were generally classified into 3 major clades: clade A (52 members) includes homologs of AtARF5, AtARF6/8, and AtARF7/19; clade B (33 members) represents the homologs to AtARF1, AtARF2, AtARF3/4, and AtARF13; and the remaining 27 ARFs belonged to clade C, which consists of AtARF16/10 and AtARF17 homologs. According to the phylogenetic tree, members of LsARFs were detected from almost all subclades, despite the fact that lettuce ARFs homologous to AtAARF17 were not identified in our analysis. Generally, our phylogenetic tree revealed that the ARF proteins from the lettuce and Solanaceae (tomato and potato) usually were clustered into the same subclades, indicating the closer relationship between the Solanaceae and Asteraceae families compared to Arabidopsis or rice.

The phylogeny of the ARF family was investigated in detail according to the previous proposed standard [24]. As illustrated in Figure 5, clade A contained five subclades represented by nodes a, b, c, d, and e, and the phylogenetic relationships within the subclades of clade A were well supported by bootstrap percentage. Node *a* (ARF6/8 subclade) subclade was placed as a twin-sister to node *b* (bryophytes ARF6/8 subclade). Node *c* was represented by ARF7/19, while node *d* included AtARF5 and its homologs. Notably, the node *e* subclade, containing class A-ARFs of bryophytes, was basal to the other four subclades, suggesting they might represent precursors of class A-ARFs. Clade B was further divided into four subclades, represented by node *f* (ARF3/4 subclade), *g* (ARF1/9 subclade), *h* (ARF2 subclade), and *i* (ARF13 subclade). Notably, class B-ARFs consisted of members only from land plants, suggesting that these higher plant-specific ARFs. Clade C included node j (ARF10/16 subclade), k (proto-C subclade), and l (ARF17 subclade). It was noted that the basal node k subclade was only represented by members from bryophytes, implying they might be a group of proto-C-ARFs.

### 2.6. Expression Profiles of LsARFs among Various Tissues and Organs

**Figure 6:**
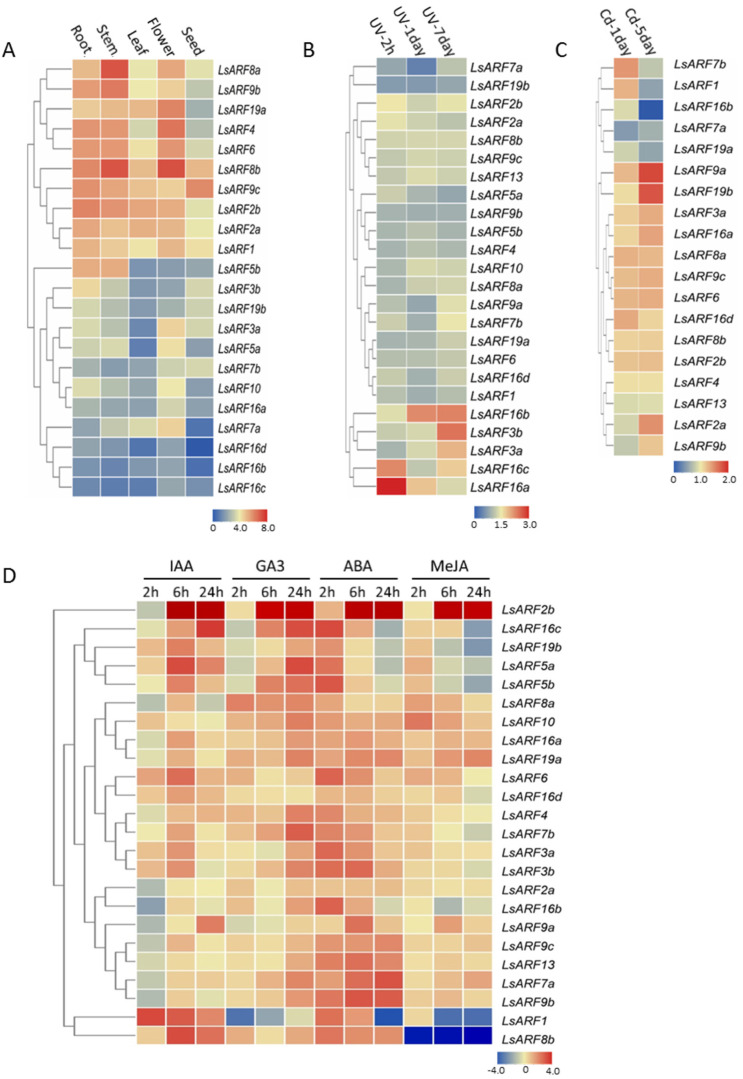
Expression profiles of *LsARF* genes. (**A**) Expression profiles of *LsARF*s with hierarchical clustering in different organs. (**B**,**C**) Heatmap representation and hierarchical clustering of *LsARF*s under UV treatments and cadmium stresses. (**D**) Heatmap showing expression patterns of the *LsARF*s under different phytohormone treatments. The FPKM value or normalized qRT-PCR data of representative transcripts of *LsARF*s was used to generate heatmap with hierarchical clustering on the basis of the Manhattan correlation with average linkage using the TBtools software package. The color scales below heatmap show the expression levels. Red indicates high transcript abundance, while blue indicates low abundance.

To determine the tissue-specific expression patterns of *LsARF* genes, we utilized the transcriptome data derived from the illumine RNA-Seq reads stored in the NCBI. The RNA-seq data provide the expression of 40,341 lettuce genes in five tissues (root, stem, leaf, flower, and seed). According to the RNA-Seq libraries, 22 members of *LsARF*s were detected in various organs and exhibited tissue-specific expression patterns, while *LsARF9a* and *LsARF13* were absent, probably due to their low transcriptional levels or spatiotemporal expression patterns. As shown in Figure 6A, *LsARF1/2a/2b/4/6/8a/8b/9b/9c/19a* were ubiquitously and highly expressed in tissues, suggesting that these ARFs might be engaged in the auxin-regulated plant growth and development in various organs. Of 24 expressed lettuce *ARF*s, *LsARF8a/8b* were the most abundant transcripts in stem and flower tissues, implying that they might be implicated in floral development such as bolting and flowering. On the contrary, some *LsARF*s such as *LsARF7a* and *LsARF16b/16c/16d* were mildly expressed (Figure 6A), suggesting these *LsARF*s might be less required in those organs. Despite low transcriptional abundance in several tissues, *LsARF7a*, as well as *LsARF3a*, was preferentially expressed in the flower tissues compared to other organs (Figure 6A), indicating that they might serve as transcriptional regulators of auxin-signaling cascade mainly in the flower development. Thus, the results suggest that these *LsARF* genes have specific functions in different organs.

### 2.7. Expression Responses of LsARFs to UV and Cd Stresses

As the auxin signaling pathway is associated with abiotic stress, we examined its expression response to abiotic stress with RNA-Seq data available in the NCBI database. As shown in Figure 6B, the transcript levels of most *LsARF* genes remained unchanged or slightly upregulated under UV treatments, suggesting that their expressions might be independent of UV stress. However, expression of *LsARF16a* and *LsARF16c* increased by 2.3- and 3.1-fold after 4 h UV treatment and remained at a relatively high level at 7-day UV treatment, suggesting they might be involved in the early response of lettuce plants to the UV stress. Transcript accumulations of *LsARF16b*, *LsARF3a*, and *LsARF3b* reached their highest levels after 7-day UV treatment, indicating that they were late-responsive genes.

Likewise, the expression of almost all *LsARF* genes could respond to the cadmium stress, except *LsARF3b*, *LsARF5a*, *LsARF10*, and *LsARF16b/16c* (Figure 6C). Of the 19 *LsARF*s, most could be induced by 3-day or 5-day cadmium stress. In particular, *LsARF9a* and *LsARF19b* exhibited a relatively higher expression in Cd-treated lettuce seedlings, which pointed to their crucial roles in plant adaption to heavy metal stress. Conversely, expressions of *LsARF7a*, *LsARF16b*, and *LsARF19a* were downregulated at 5-day cadmium treatment, indicating they might be negative regulators of auxin-related signaling pathways under heavy metal stress. Thus, the results suggest that the LsARF-mediated auxin cascade might contribute to cadmium stress.

### 2.8. Expression of LsARFs in Response to Various Phytohormone Treatments

The expression changes of *LsARF*s under different phytohormone or chemical analog treatments by the qRT-PCR analysis were evaluated in the leaf tissues of one-month-old seedlings. In general, compared to the controls, the majority of lettuce ARFs considered in the study were upregulated by ABA, GA3, or IAA treatments (Figure 6D). In auxin treatments, *LsARF1* expression was activated at 2h-IAA treatment, but its expression reduced gradually, suggesting it might be early responsive genes; expression of most LsARF genes peaked at 6h- or 24hIAA treatments, indicating that they could be involved in the plant late responses to auxin; by contrast, LsARF8a expression was repressed by auxin treatments, implying its negative roles in auxin signaling pathways. Moreover, expression levels of several *LsARF*s were kept unchanged in auxin-treated leaves, suggesting that they were insensitive to auxin. It is worth noting that ABA could stimulate the expression of many *LsARF* genes, and their expression peaks were reached mainly at 2h-ABA treatments. The results are consistent with the findings that ABRE cis-elements were identified in the promoter regions of most *LsARF*s. Almost all *LsARF*s were gibberellin-responsive genes, while their expressions were elevated to the maximum probably after 24-h GA3 treatments. In contrast, methyl jasmonate had minor effects on the expression of *LsARF* genes, despite the fact that a few *LsARF*s including *LsARF2b*, *LsARF10*, and *LsARF19* could be triggered upon the MeJA treatments. Together, these results provide experimental evidence that ARFs play central roles during the interplay between diverse phytohormone signaling pathways. 

### 2.9. Gene Expression Analysis of LsARFs under Thermal Treatments

**Figure 7:**
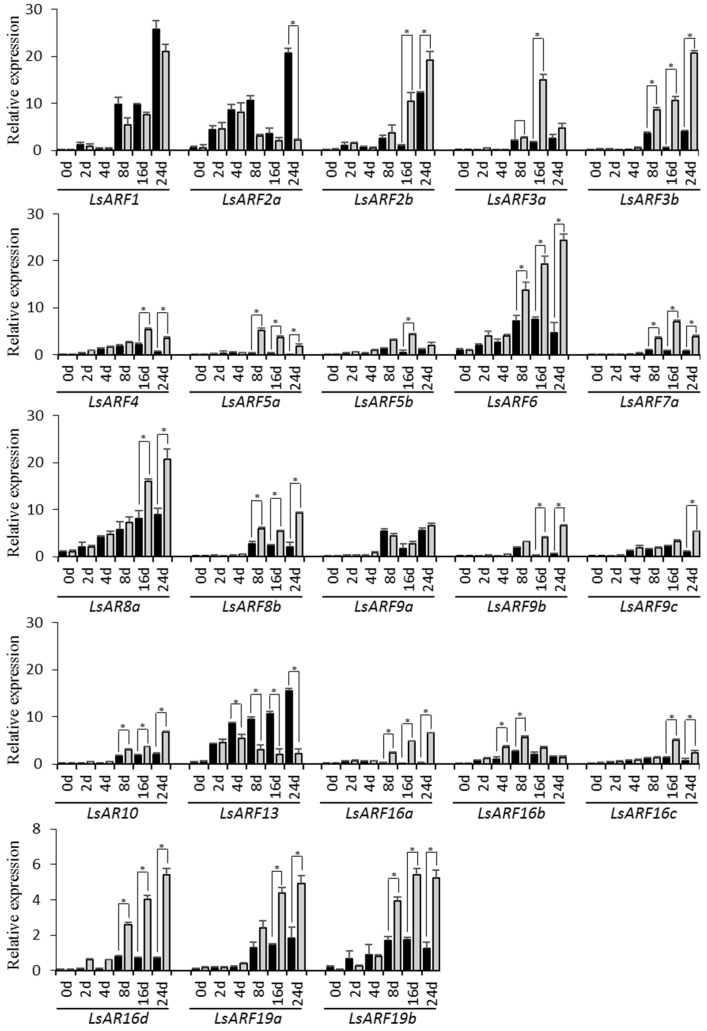
qRT-PCR analysis of *LsARF* genes in response to heat treatments. *LsARF* transcript levels measured by real-time RT-qPCR using cDNAs from the shoot apex at indicated time points. Data are means of three biological replicates (8 pooled plants each), and error bars denote SE. Lettuce *eIF4ɑ* gene was used as an internal control. Stars above the error bars indicate significant differences between treatments and the mocks (according to Student’s *t*-test, *p* < 0.05). qRT-PCR primers for each *LsARF* gene are provided in Table S1.

To explore their association with thermally induced bolting, we examined the expression levels of *LsARF* genes in the stem tissues of lettuce plants exposed to heat treatment for 0, 2, 4, 8, 16, and 24 days (Figure 7). As predicted, transcripts of most *LsARF* genes were accumulated to high levels during the bolting of lettuce plants. Among them, expression levels of *LsARF1*, *LsARF2a/2b*, *LsARF8a*, and *LsARF13* were dramatically increased with the elongation of the stem within 24 days, while other *LsARF* genes, including *LsARF3a/3b*, *LsARF6*, *LsARF9a*, and *LsARF16b*, displayed a mild increase in transcript accumulations compared with their controls, which implies that they could be actively involved in the floral transition such as bolting. In contrast, several *LsARF*s such as *LsARF5a/5b*, *LsARF7a*, *LsARF9b/9c*, and *LsARF16c/16d* did not show any significant changes in expression levels, indicating that they might not be required for bolting under normal growth conditions.

High temperature has a profound impact on the expression patterns of *LsARF* genes (Figure 7). Transcript levels of the majority of *LsARF* genes were induced by thermal stress, including *LsARF2b*, *LsARF3a*, *LsARF6*, *LsARF8a*, and *LsARF16a*. Conversely, high temperature significantly decreased the expression of many *LsARF*s such as *LsARF2a* and *LsARF13*. Thus, those *LsARF*s with up- or downregulated expressions in response to high temperatures might mediate the expression of their downstream genes in the auxin signaling pathways, ultimately leading to developmental alternation upon warm temperatures. In addition, the expression profiles of *LsARF1*, as well as *LsARF9a*, were almost identical between heat-treated and control samples, suggesting that they might be insensitive to thermal stimuli.

### 2.10. LsARF8a Encodes an ARF Transcription Factor

**Figure 8:**
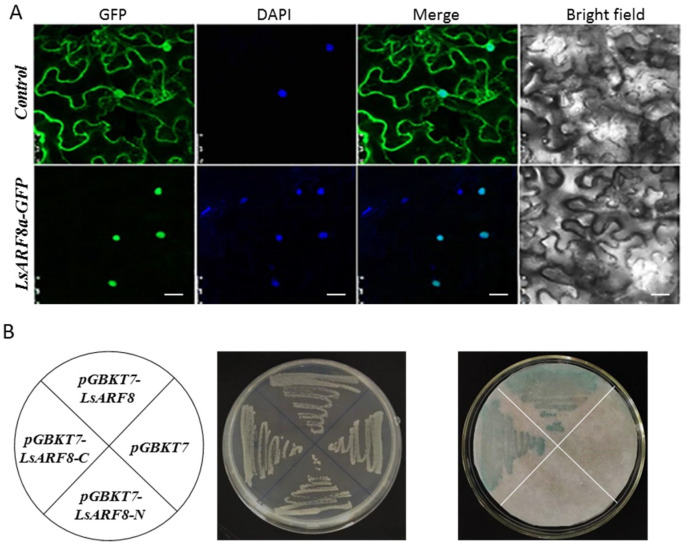
*LsARF8a* encodes a transcriptional activator. (**A**) Subcellular localization of LsARF8a-GFP fusion proteins. Indicated constructs were agro-infiltrated into *N. benthamiana* leaves, and the subcellular localization of GFP alone (upper panel) or LsARF8a-GFP fusion proteins (lower panel) was analyzed by confocal laser-scanning microscopy. Images of representative leaf epidermal cells were taken 48 h after agro-infiltration. The photographs (left to right): bright field, GFP fluorescence, DAPI fluorescence, and merge. Bar = 20 μm. (**B**) Transcriptional activity analysis of LsARF8a in yeast cells. *pGBKT7-LsARF8a*, pGBKT7-*LsARF8a-N* (1–385 AA), *pGBKT7-LsARF8a-C* (386–799 AA), and pGBK&7 vector were transformed into yeast strain AH109 (left); the transformants grew well on SD/-Trp plate (middle), and colony-lift filter assay showed their β-galactosidase activities (right).

According to the gene expression analysis, *LsARF8a* exhibited much higher expression in stem tissues, and importantly, its expression was strongly repressed by high temperatures during the floral transition stage. Therefore, *LsARF8a* might be tightly associated with the elongation of stem stalk in lettuce, and its possible role during thermally induced bolting was further investigated. Sequence analysis identified a nuclear localization signal in the N-terminal region of the putative LsAR8a protein, consistent with its predicted role as a transcription factor. To test the possibility, we transiently expressed *LsARF8a-GFP* in *N. benthamiana* leaf epidermal cells by infiltration with *A. tumefaciens* harboring a construct designed to express the *LsARF8a* ORF N-terminally fused to GFP. As revealed by the microscopy, GFP-labelled LsARF8a proteins were observed only in the nucleus, while free GFPs were distributed throughout the nucleus and the cytoplasm (Figure 8A). The results indicated that LsARF8a could redirect the GFP from the cytosol to the nucleus, supporting its function as a transcription factor.

To test the transcriptional activity of LsARF8a, the fusion plasmids *pGBKT7-LsARF8a*, pGBKT7-*LsARF8a-N*, and *pGBKT7-LsARF8a-C* were separately transformed into yeast strain AH109, and *pGBKT7* vector was used as the negative control. The transformed yeast strains grew well on the synthetic dextrose (SD) medium lacking tryptophan (SD/-Trp) but containing 20 mM 3-AT. The β-galactosidase activity assay suggested that *LacZ* expression in the yeast cells harboring *pGBKT7-LsARF8a* or *pGBKT7-LsARF8a-C* was transactivated by LsARF8a or its C-terminal alone, yet no significant expression could be detected in those yeast strains carrying *pGBKT7*-LsARF8a_C or *pGBKT7* empty vector (Figure 8B). Thus, the results demonstrated that N-terminal polypeptide confers LsARF8a in the transcriptional activation activity in yeast cells. Hence, the results suggest that LsARF8 might be a functional transcriptional factor in lettuce.

### 2.11. Silencing of LsARF8a Resulted in Delayed Bolting under Thermal Treatments

To investigate the roles of LsARFs during lettuce bolting, we selected *LsARF8a* for functional analysis by the virus-induced gene silencing (VIGS) method. A 250 bp fragment targeting the *LsARF8a* 5′-region was chosen for the insertion into the viral vector *pTRV2* vector, and the resulting plasmid *pTRV2-LsARF8a* was introduced into Agrobacterium. The Agrobacterium lines harboring *pTRV2-PDS* and empty *pTRV2* vector served as controls. Lettuce seedlings with four true leaves were transformed by leaf injection with the aforementioned Agrobacterium as well as the prepared *pTRV1* agrobacterium lines. After a three-week recovery under normal growth conditions, all silencing lines were verified by the qRT-PCR method. We found that leaves of *pTRV2-PDS* lines exhibited photo-bleaching phenotypes, which agreed with the reduction of *PDS* genes. qRT-PCR analysis with leaf tissues suggested that transcript accumulation of *LsARF8a*, compared to the control plants, significantly decreased in *LsARF8a-*sliciencing lines, whereas expression of *LsARF6* and *LsARF8b* was not affected (Figure S3), suggesting that *LsARF8a* was specifically silenced. Subsequently, half *LsARF8*-knockdown (*TRV:LsARF8a*) plants were transferred to another growth chamber for heat treatments, and lettuce plants transformed with *pRTV2* empty vectors (*TRV:00*) and WT plants were used as controls.

**Figure 9:**
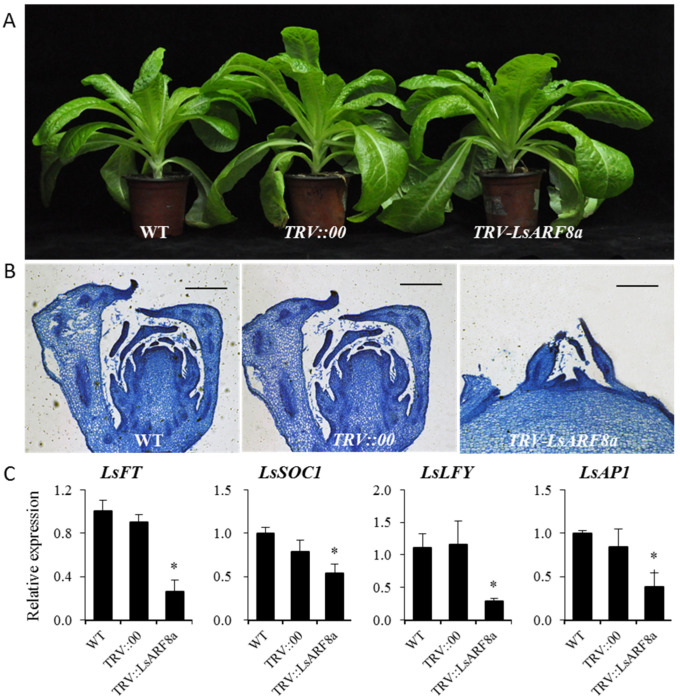
Functional characterization of *LsARF8a* during the thermally induced bolting in lettuce. (**A**) Phenotypic characterization of *TRV2-LsARF8a*, *TRV:00*, and WT lettuce plants after one-month heat treatments. (**B**) Microscopic observation of shoot apices from *TRV:LsARF8a*, *TRV:00*, and WT lettuce plants after one-month thermal stress. Bar = 200 μm. (**C**) qRT-PCR analysis of *LsFT*, *LsSOC1*, *LsAP1*, and *LsLFY* in *LsARF8a*-silencing and control plants. Error bars indicate SE from three biological replicates, and asterisks indicate statistically significant differences between WT and treatments, as determined by Student’s *t*-test (* *p* < 0.05).

After growth in the thermal chamber for about one month, we assessed the responses of these lettuce lines upon heat treatments. After heat treatments, the stems of the *LsARF8a*-silencing plants were still immersed among the rosette leaves, whereas the elongated inflorescence stems emerged in the control groups (Figure 9A). Moreover, the stem length of LsARF8a knockdown lines was significantly lower than that of WT, whereas there was no difference between WT and *TRV::00* lines, suggesting that silencing of *LsARF8a* could suppress stem elongation under thermal conditions (Figure S4). Moreover, the anatomical comparisons of the stem apex (inflorescence meristem) were conducted using these one-month heat-treated plants. As illustrated in Figure 9B, the stem apex of *LsARF8a*-silenced plants became much larger and more flattened, indicating the initial stage of floral differentiation; on the contrary, flower bud differentiation was observed in these non-silenced lines (*TRV:00* or WT plants), suggesting that their floral transitions were completed after one-month heat treatments. In addition, it is noticeable that, under control temperatures, the bolting time of *LsARF8a* silencing lettuce plants was delayed significantly compared to the control groups, indicating that absence of *LsARF8a* led to the late bolting under normal conditions. Together, the results suggest that downregulation of LsARF8a resulted in the delayed bolting, thus supporting the fact that LsARF8a might positively regulate plant bolting in lettuce.

Next, we still needed to fill up the lacuna on the identification of downstream genes of LsARF8a in floral transition signaling pathways. As some genes such as *LsFT, LsLFY*, LsAP1, and *LsSOC1* have been reported to be the inducers that are involved in the lettuce bolting [25,26,27], their expression profiles were investigated in the *LsARF8a*-silencing lines through qRT-PCR analysis. As shown in Figure 9C, we found that the expression levels of *LsFT, LsLFY*, and *LsSOC1* were downregulated in the *LsARF8a*-silencing lettuce. The results indicate that LsARF8a might be a positive regulator to activate these floral activator genes directly or indirectly, thereby accelerating plant bolting in lettuce.

## 3. Discussion

### 3.1. Expansion of Lettuce ARF Gene Family

As the major effector of numerous aspects of auxin responses, ARF transcription factors translate the chemical signal into the transcriptional regulation of a defined set of genes. Due to their functional importance in the auxin cascades, the ARF gene family has been investigated intensively in the model plant Arabidopsis and to varying degrees in non-model plant species. Previous studies revealed that the Arabidopsis genome contains 23 ARF genes, while 25, 51, and 36 ARF members were identified in rice, soybean, and corn genomes [28,29,30], respectively. The genome size of lettuce is 2.5 Gb, which is one of the largest plant genomes assembled thus far [22]. Considering that lettuce has such a large genome, it was assumed that the lettuce genome could encode a larger ARF gene family as previously reported in soybean and corn [29,30]. Intriguingly, only 24 ARFs were finally identified from the lettuce genome, indicating that ARF transcription factors might be encoded by a moderate-sized gene family. Although the lettuce genome is nearly 17 times larger than the Arabidopsis counterpart, the numbers of the ARF gene family in the two genomes are still comparable, which contradicted the genome complexity between the two species. It is noteworthy that Arabidopsis has seven members of the ARF13 subclade while only one belongs to the subclade. It is plausible that several independent, small-scale, segmental duplication events and chromosome rearrangements that occurred at *ARF13* loci resulted in multiple members of the AtARF13 subclade, which led to the expansion of the ARF gene family in Arabidopsis.

Furthermore, we investigated the distribution of lettuce and Arabidopsis *ARF* members in different subclades. With respect to the class A-ARFs, five *LsARF*s belong to the ARF10/16 subclade, while only two *AtARF* genes were grouped into this subclade; surprisingly, lettuce ortholog to *LsARF17* was absent from the ARF17 subclade, whereas its counterparts could be found in rice, tomato, and potato. Despite the lack of ARF17 ortholog in lettuce, lettuce *ARF16/10* subclade expanded in class A-ARFs due to the duplications of *LsARF16*s. Similarly, duplication events in the lettuce genome were observed in class B- and class C-ARFs, except in the ARF13 subclade. Hence, we conclude that the total number of *AtARF* genes is smaller than that of *LsARF*s. There is a whole-genome triplication event in lettuce since the divergence from the grape lineage and the triplicated regions cover at least 26% of the lettuce genome [22,31]. Consequently, genes encoding some but not all transcription factors and DNA-binding proteins were enriched in the triplicated regions of the lettuce genome [22]. Therefore, it is reasonable that some *LsARF*s might be present in these duplicated regions and that their duplicated copies contribute to the expansion of the ARF gene family in the Asteraceae.

With the expansion of the lettuce ARF family, functional redundancy and divergence could occur among different *LsARF*s. Phylogenetic analysis suggested that *LsARF5a* and *LsARF5b* form sister pairs within the ARF5 subclade, and thereby they might execute similar functions in auxin-regulated pathways. Despite the lack of experimental evidence, the expression profiles of two *LsARF5*s were almost identical in the respective hormone treatments, supporting the assumption that they might play overlapping roles in these signaling pathways. However, this is not always the case. It seems that subfunctionalization is common for *LsARF*s in the same subclade. For example, *LsARF2a* and *LsARF2b* genes displayed contrasting expression behaviors in response to various phytohormone applications. The qRT-PCR analysis revealed that *LsARF2a* was insensitive to all phytohormones, whereas *LsARF2b* was highly inducible genes upon ABA, auxin, gibberellin, or MeJA. Given that the expression profiles differ across individual members of the *LsARF16* subclade, their functions might have diverged considerably. Similar assumptions could be applied to other *LsARF* members that were grouped in the same subclade. Together, our findings reflected that, in most cases, *LsARF* members from the same subclade might have acquired their unique roles.

### 3.2. LsARFs Are Involved in the Heat-Induced Bolting

Plant exposure to high temperatures causes diverse developmental, physiological, and morphological responses, including early flowering, accelerated shoot and root growth, and changes in stomatal differentiation [32,33,34,35]. Nevertheless, the signaling pathways of how plants sense and respond to elevated temperatures are incompletely understood. Recent studies have demonstrated that PHYTOCHROME B (PHYB) perceives the thermal signals and therefore connects plant growth and ambient temperatures [36,37]. PHYB exists in two relatively stable forms: a red-light-absorbing inactive Pr form and the far-red-light-absorbing active Pfr form, and the Pfr form can be converted into Pr form through a temperature-dependent process called thermos-reversion. In Arabidopsis seedlings, the phytochrome null mutants (*phyABCDE*) lacking phytochrome activity exhibit constitutive high-temperature responses such as elongated hypocotyl, which resemble the plant behavior under heat treatments [36]. Interestingly, it is worth noting that a small number of genes associated with auxin signaling and elongation growth including *YUCCA8*, *YUCCA9*, *LIKE AUXIN RESISTNAT1*, *TRANSPORT INHIBITOR1*, *ARF7*, and *ARF9* are upregulated in the mutant background, which argues in favor of crucial contributions of auxin signaling during plant heat responses [36]. In previous experiments, we have shown that exogenous application of auxin could promote early bolting, albeit blocking of auxin transport with TIBA (2,3,5-triiodobenzoic acid) led to the delayed bolting in lettuce, which is in agreement with the hypothesis that auxin signaling is needed in lettuce bolting [16,38]. Additionally, we demonstrated that “loss-of-function” of *LsARF3* led to the delayed bolting in lettuce under heat-treated or control conditions, further indicating that auxin signaling allows for lettuce bolting [35]. Consistent with the aforementioned findings, we further suggested that most *LsARF*s, the central components of the auxin signaling pathways, displayed altered expression profiles upon heat treatments, implying their contribution to the warm temperature-induced bolting. Therefore, our results further showed that ARF-mediated auxin signaling is responsible, at least partially, for these altered phenotypes upon heat treatments.

### 3.3. LsARF8a Postively Regulates Lettuce Bolting under Thermal Growth Conditions

Accelerated bolting is regarded as the hallmark of lettuce responses to thermal conditions [19], being regulated by an elaborate network of genetic pathways responsive to endogenous and external signals. The ambient temperatures accelerate the thermos-reversion rate of the PHYB, leading to a reduction of the steady-state level of the active Pfr. Because the active Pfr can repress the transcription of a group of bHLH transcriptional factors such as PHYTOCHROME-INTERACTING FACTOR4 (PIF4) and PIF5, thermal treatment leads to the induction of *PIF4* at the transcriptional levels in Arabidopsis [39]. PIF4 directly regulates the expression of a defined set of growth-relevant genes, particularly those genes in auxin biosynthesis and signaling [40,41]. In our study, promoter analysis of *LsARF8a* identified two G-box (5′-CACGTG-3′) motifs that could be recognized by bHLH proteins [42], suggesting LsARF8 might be the direct target of PIF4. Intriguingly, G-box motifs were also found in the promoter regions of other 15 *LsARF*s, expression profiles of which were largely altered upon warm temperatures. For example, the promoter regions of *LsARF3a*, *LsARF3b*, and *LsARF13* contain two, two, and three G-box motifs, respectively. Thus, the results suggest that more than half *LsARFs* could be dynamically regulated by PIFs in a temperature-dependent manner.

In addition, it is unusual that there are so many *ABRE* cis-elements in the promoter regions of *LsARF8a* and other 16 *LsARF*s, implying that their expressions were possibly influenced by the *ABRE*-binding protein (AREB)/ABRE-binding factor (ABF) transcription factors. It was revealed that *AREB1/ABF2*, *AREB2/ABF4,* and *ABF3* redundantly control a major part of ABA-mediated transcriptional changes when plants encounter dehydration, cold, heat stresses, or other abiotic stresses [43,44]. qRT-PCR demonstrated that ABA could induce the expression of *LsARF8a*, which is in line with the cis-element analysis of its promoter regions. Taken together, PIF4, as well as ABRE/ARFs, could play equally important roles in stimulating the transcription of *LsARF*s such as *LsARF8a* under higher temperatures. The possible scenario is that the transcription of *LsARF8a* might be modulated either by the ABREs solely or by the PHYB-PIF4 node upon heat treatments.

The silencing of *LsARF8a* resulted in late flowering in warmer conditions, thereby suggesting that LsARF8a contributed to lettuce bolting. Furthermore, *LsARF8a* was preferentially expressed in stem tissues, supporting its potential role in stem development. On the basis of these observations, we propose that LsARF8a might positively regulate the floral transition process in lettuce. In particular, *LsARF8a* transcripts in the shoot apex accumulated with the transition from the vegetative to the floral stage, which indicated that LsARF8a is required for lettuce bolting and flowering under normal growth conditions. Given that LsARF8a is a transcription activator, these results imply that LsARF8a could promote plant bolting by stimulating the expression of the floral gene(s) during the early stage of floral transition. Several floral activators such as LsFT, LsMADS55, LsLFY, LsAP1, and LsSOC1 have been reported in lettuce [25,26,27,45]. Consistent with this hypothesis, we found an associated reduction in *LsFT*, *LsSOC1*, and *LsLFY* transcript levels in the *LsARF8a* knockdown lines. It is possible that the downregulation of these floral inducers due to the *LsARF8a* silencing is responsible for the delayed bolting and flowering. Therefore, our observations from LsARF8a might indicate the existence of a previously unknown mechanism whereby thermal stress regulates the timing of plant bolting via heat-induced, ARF-mediated floral signaling pathways. However, it is still unknown as to how LsARF8a controls the floral genes to coordinate the timing of lettuce bolting, especially during thermal conditions. Hence, our future work will be focused on the systematic identification of direct targets of LsARF8a through comparative transcriptome analysis of *LsARF8a* overexpressing and knockout lines. We are generating these stable *LsARF8a* transgenic lines that will be an impressive feat to better understand the LsARF8a-mediated bolting in lettuce.

## 4. Materials and Methods

### 4.1. Plant Material and Treatments

Experiments were performed in a greenhouse located at the National Engineering Research Center for Vegetables (Beijing, China). The seeds of leafy lettuce (*Lactuca sativa* L.) varieties GB-30 were provided by the Beijing University of Agriculture. Seeds were sown in the plug trays, using Jeffery perlite as the substrate, in a growth chamber. One-week-old seedlings with uniform height were transplanted to the greenhouse, enabling only one plant per pot, and the growth conditions are as follows: 16 h day, 20 °C/8 h night, 13 °C. The lettuce seedlings were grown until the fourth leaf was fully expanded. For heat treatment, lettuce plants were transferred to another greenhouse, and the growth conditions were changed to 16 h day, 33 °C/8 h night, 25 °C. The remaining plants grown in normal conditions were used as controls. For phytohormone treatments, lettuce plants with four true leaves were subjected to IAA (100 μM), ABA (50 μM), GA3 (10 μM), or MeJA (10 μM). After phytohormone treatments, the leaf tissues were collected at designated points and immediately frozen in liquid nitrogen for RNA extractions. Sample collections were performed on separate days for the replicates.

### 4.2. Identification of LsARF Genes from the L. sativa Genome

To investigate the *ARF* gene family in the lettuce genome, all members of *ARF* sequences from *Arabidopsis thaliana* and rice were used as querie sequences for a BLAST search against Phytozome (https://phytozome.jgi.doe.gov/, accessed on 19 July 2022) with default parameters [46]. These candidates were verified the presence of the B3 (PF02362), AuxRE (PF06507), and PB1 (PF00564) domain using SMART (http://smart.embl-heidelberg.de/smart/batch.pl, accessed on 25 July 2022) and CDD-search (https://www.ncbi.nlm.nih.gov/Structure/bwrpsb/bwrpsb.cgi, accessed on 25 July 2022). The redundant LsARF sequences were manually removed by multiple sequence comparisons. Finally, the non-redundant LsARF sequences were used as queries to blast against the Phytozome database. The representing gene model for each *LsARF* locus was identified, and their corresponding information on chromosomal location, locus ID, and transcript ID were obtained simultaneously.

### 4.3. Analysis of Gene Structure and Conserved Domains

On the basis of the genome annotation available in Phytozome, the intron–exon structure of individual *LsARF* genes was predicated, and its genomic organization was visualized using the Gene Structure Display Server [47]. Conserved domains in protein sequences were verified using ScanProsite (http://pro-site.expasy.org/scanprosite/, accessed on 30 July 2022), which provides information about the positions of different domains in the protein sequence. The MEME program (https://meme-suite.org/meme/tools/meme, accessed on 19 July 2022) was used to predict the novel motifs with the default settings. This information was visualized with TBtools to show the representation of the distribution of domains in the deduced amino acid sequences of LsARF proteins [48].

### 4.4. Sequence Alignment and Phylogenetic Construction

Multiple alignments of LsARF protein sequences from *Marchantia polymorpha*, *Physcomitrium patens*, *Solanum lycopersicum*, *Solanum tuberosum*, *Arabidopsis thaliana*, *Oryza sativa*, and *Lactuca sativa* were conducted using ClustalW [49]. The neighbor-joining method was used to conduct a phylogenetic analysis in MEGA X, with 500 bootstrap replicates [50].

### 4.5. Expression Profiling of LsARF Genes in Different Tissues or under Various Stresses

The lettuce RNA-Seq data were downloaded from the Gene Expression Omnibus (GEO) in the NCBI (GSE105821, GSE143675) or Genome Sequence Archive in National Genomics Data Center (PRJCA007442) databases, and the corresponding FPKM (fragments per kilobase per million reads) values for *LsARF*s were obtained for five organs representing major organs including root, stem, leaf, flower, and seed. As described, abiotic-stress-treated samples include lettuce plants exposed to UV (2h and 4h) or cadmium stress (3 days and 5 days). Similarly, FPKM values for abiotic-stress-treated lettuce plants were calibrated by calculating the fold change of expression levels between treatments and the corresponding controls. The normalized expression data were used to generate heatmaps by using the TBtools software package [48].

### 4.6. RNA Extraction and Quantitative Real-Time PCR

Total RNA was extracted with an EasyPure Plant RNA Kit (Transgene, Beijing, China). RNA quantity and quality were assessed using a NanoDrop8000 (Thermo Scientific, Waltham, MA, USA). Total RNA isolation and reverse transcription with oligo (dT)_18_ were performed as described previously [51,52]. The amounts of individual genes were quantified with gene-specific primers by real-time PCR analysis with a LightCycler 480 System (Roche, Rotkreuz, Switzerland) and SYBR Green mixture (Toyobo, Shanghai, Japan). The relative expression of specific genes was quantitated with the 2^−∆∆Ct^ calculation method, where ΔΔCt is the difference in the threshold cycles and the reference housekeeping gene [53]. Lettuce *eIF4A* was used as the reference gene for normalizations, and the sequences of specific primers are shown in Table S1.

### 4.7. Subcellular Location of LsARF8a

The cDNA fragment containing the coding region of LsARF8a was fused to the 5′-end of the GFP coding region, and the fused gene was subcloned into *pRI101* under the controls of the 35S promoter through homologous recombination. The resulting plasmid pRI101-LsARF3 and the pRI101 vector were transformed into *Agrobacterium tumefaciens* strain GV3101 and infiltrated into tobacco (*Nicotiana benthaminana*) leaves. The plants were incubated in the dark at 24 °C for one day and then transferred to the greenhouse with a 16 h light/8 h dark photoperiod for an additional day. Nucleus DNA was counterstained with DAPI, and cells were visualized with excitation at 405 nm (DAPI) and 488 nm (GFP) under a confocal microscope.

### 4.8. In Vivo Transcriptional Activity Assay

The full-length coding region, 5′-fragment (ATG-1155), and 3′-fragments (1156–2397) of *LsARF8a* were fused to the *GFP* under the control of the 35S promoter, and the resulting plasmids *pGBKT7-LsARF8a-N* and *pGBKT7-LsARF8a-C* were transformed into yeast strain AH109, as described previously [54]. The pGBKT7 vector was used as a control. These yeast transformants were screened on the synthetic dextrose (SD) medium without histidine, leucine, and uracil. *β*-Galactosidase activities of these yeast transformants were examined according to the guidance in the Yeast Protocols Handbook (Clontech, Mountain View, CA, USA).

### 4.9. Virus-Induced Gene Silencing (VIGS)

*pTRV2-LsARF8a* was generated by cloning a PCR-fragment-amplified lettuce leaf cDNA template using specific oligonucleotide primers incorporating Sal I and Xba I restriction sites, respectively, at the 5′- and 3′-ends for cloning into virus vector *pTRV2*. The *Agrobacterium tumefaciens* strain GV3101 harboring the recombinant plasmids *pTRV2-LsARF8a* and *pTRV1* were used in vitro agroinoculation by leaf-injecting of one-month-old lettuce plants. The Agrobacterium lines carrying the *pTRV2* empty vector or *pTRV2-PDS* were used as negative and positive controls, respectively. Primers used for vector constructions are listed in Table S1.

## 5. Conclusions

In this study, 24 *LsARF* genes were identified from the lettuce genome. The phylogenetic relationship, chromosomal distribution, exon–intron structure, consensus motifs, domain compositions, and promoter analysis provide valuable insights into the evolutionary aspects of the *LsARF* gene family. Expression analysis suggested that *LsARF* is dynamically involved in diverse biological processes, including organ development, phytohormone, and responses to abiotic stimuli. Subcellular location and transcriptional activity analysis suggested that *LsARF8a* might encode an ARF transcriptional factor. VIGS analysis indicated that LsARF8a might be a positive regulator of accelerated bolting under thermal conditions. In addition, G-box and ABRE cis-elements were identified in the promoter regions of *LsARF8a* and other *LsARF*s, implying that PhyB and ABRE might control the expression of *LsARF* genes, respectively. On the basis of the previous findings in Arabidopsis and our results, we propose a novel molecular mechanism explaining the thermally accelerated bolting: Warm temperatures stimulate the conversion to inactive Pr, which releases the depression of PIF4 and other PIFs; the transcript accumulations of PIFs as well as heat-inducible ABREs/ABFs facilitate the activation of *LsARF8a*, resulting in the activation of floral activator genes including *LsFT*, *LsLFY*, and *LsSOC1* and thus promoting stem elongation in lettuce. These findings expand our knowledge of the *LsARF* gene family, and importantly, identified the positive role of LsARF8a in the thermally induced bolting in lettuce, which will facilitate the genetic improvement of heat tolerance in horticultural crops.

## Figures and Tables

**Table 1:** List of putative the gene family of auxin response factors in the *L. sativa* genome.

Gene Name ^a^	Locus ID ^b^	Chromosomal Location ^c^				Gene Models ^d^	Putative Proteins ^e^		
		Chr	Chr_Start	Chr_End	Direction		Length (aa)	pI	MW (kDa)
*LsARF1*	Lsat_1_v5_gn_7_74681	7	123854596	123861992	F	2	680	5.64	76.25
*LsARF2a*	Lsat_1_v5_gn_1_32200	1	34835165	34839317	R	2	731	6.50	81.82
*LsARF2b*	Lsat_1_v5_gn_5_9980	5	20298945	20303345	F	2	789	6.07	88.69
*LsARF3a*	Lsat_1_v5_gn_7_22140	7	30074288	30078405	R	3	610	6.94	67.76
*LsARF3b*	Lsat_1_v5_gn_4_51680	4	76542160	76545742	F	2	432	7.07	47.53
*LsARF4*	Lsat_1_v5_gn_2_98041	2	177742824	177747158	F	2	786	6.19	86.48
*LsARF5a*	Lsat_1_v5_gn_9_46321	9	51222149	51227761	F	2	732	5.30	81.55
*LsARF5b*	Lsat_1_v5_gn_2_12381	2	29392285	29399177	F	2	838	5.32	92.71
*LsARF6*	Lsat_1_v5_gn_7_27300	7	36888567	36894316	F	3	811	5.91	89.00
*LsARF7a*	Lsat_1_v5_gn_5_139420	5	267927916	267933344	F	2	1025	6.26	115.76
*LsARF7b*	Lsat_1_v5_gn_7_35261	7	49118344	49120839	F	1	588	5.56	66.34
*LsARF8a*	Lsat_1_v5_gn_3_68721	3	90790977	90795537	F	2	799	5.78	88.99
*LsARF8b*	Lsat_1_v5_gn_2_72540	2	146432295	146436809	F	2	845	5.88	94.28
*LsARF9a*	Lsat_1_v5_gn_6_9480	6	16055386	16059645	R	2	532	6.45	60.34
*LsARF9b*	Lsat_1_v5_gn_6_84121	6	138245572	138249925	R	2	662	6.22	74.49
*LsARF9c*	Lsat_1_v5_gn_3_11900	3	16105592	16113929	F	4	601	5.51	67.73
*LsARF10*	Lsat_1_v5_gn_2_100181	2	176445550	176448727	F	2	666	6.36	73.73
*LsARF13*	Lsat_1_v5_gn_9_2121	9	5141158	5146712	R	3	510	7.27	56.91
*LsARF16a*	Lsat_1_v5_gn_9_69060	9	88467753	88471738	R	2	530	7.65	59.46
*LsARF16b*	Lsat_1_v5_gn_2_112381	2	191567478	191570351	F	2	645	6.08	71.84
*LsARF16c*	Lsat_1_v5_gn_8_20161	8	24852532	24855669	F	2	641	7.30	72.31
*LsARF16d*	Lsat_1_v5_gn_6_30741	6	39665361	39668043	F	1	639	7.93	70.34
*LsARF19a*	Lsat_1_v5_gn_5_81601	5	179930026	179937998	R	2	990	6.12	109.53
*LsARF19b*	Lsat_1_v5_gn_7_57521	7	81907809	81916387	R	1	947	6.01	104.41

^a^ Name referred to systematic designation to members of the *LsARF* family according to the homology against *Arabidopsis*. ^b^ Gene accession number in Phytozome database. ^c^ Chromosomal location of the *LsARF* genes in the Lettuce genome (V5.0). ^d^ Isomer numbers. ^e^ Length (number of amino acids), molecular weight (kilodaltons), and isoelectric point (pI) of the deduced polypeptides were calculated using Lasergene Molecular Biology Suite (Version 7.0).

## Data Availability

Not applicable.

## Supplementary Materials

The following supporting information can be downloaded at: https://www.mdpi.com/article/10.3390/ijms232113509/s1.
