# Supplementary material for: Genome-Wide Analysis of Auxin Response Factors in Lettuce (Lactuca sativa L.) Reveals the Positive Roles of LsARF8a in Thermally Induced Bolting
Source: Int J Mol Sci. 2022 Nov 4;23(21):13509. doi: 10.3390/ijms232113509 (PMC9653848; doi:10.3390/ijms232113509)
Supplement: Supplementary file 1 [file ijms-23-13509-s001.zip › ijms-1946581-supplementary.pdf]

Supplementary Table S1. Primers used in qRT-PCR experiments.

Primers	Sequence (5'→3')	
Lsa13	CACTTCTCCCCCAACTTCAA	LsARF6
Lsa14	GCAGAGCGAGGGATGTTAAG	
Lsa15	TCCCATCATGTTCGACTTCA	LsARF19b
Lsa16	CCAAAAAGGACGGATTTTGA	
Lsa17	CGATCCTCAGCTTCATCTCC	LsARF1
Lsa18	AGCCTCAAGCTGTTCCATGT	
Lsa19	AGAAGCTTGTGTGCGGAGAT	LsARF3a
Lsa20	TTGACCACATTCGCAAAGTC	
Lsa21	ATCTTGCCACCTGATGCTCT	LsARF7b
Lsa22	ACCCCATTCCCTAAAACACC	
Lsa23	GAGGAGGACAAATCGACCAA	LsARF8a
Lsa24	TCCCAAACATTTCACCCAAT	
Lsa25	ACCAAGGTTCAAATGCAAGG	LsARF9c
Lsa26	CACAATTTCCCATTGGTTCC	
Lsa27	ACTCGATTACACCGCAGACC	LsARF16a
Lsa28	TGAGCTTCTTGTGGTTCACG	
Lsa29	ACTCATCAAATGCCGGAAAC	LsARF13
Lsa30	ACCCAAACATCCTGTCAAGC	
Lsa31	CGATTGCAGTGTTGGATGAG	LsARF5a
Lsa32	GGAGTATGCCCGAGAATCAA	
Lsa33	TATGATTCTCCGGCCATCTC	LsARF3b
Lsa34	TCACCCCATTTCTTCTCCAG	
Lsa35	GATGATCCATGGCAGGAGTT	LsARF9a
Lsa36	CAGCAGCCTCCAAGCTAAAC	
Lsa37	TGGGAAGGTGAAAGTGGAAG	LsARF16d
Lsa38	GATCTGCATTGCGTTCTTCA	
Lsa39	CTTCGAAAGCATGCAAATGA	LsARF9b
Lsa40	TGTTAGCAAATGCCTTCGTG	
Lsa41	GGTGACATGATGCTTGTTGG	LsARF2a
Lsa42	TGCCTTCTGCAACTGATGAC	
Lsa43	CAAAACGTGAAGCGAGTCAA	LsARF16c
Lsa44	GCTCCCTGAATGCTCGTAAG	
Lsa45	GTTTCTGTTGGGATGCGTTT	LsARF8b
Lsa46	TGATTCATCCCATCCAACCT	
Lsa47	CCGAAACAATTTTTCCGAGA	LsARF16b
Lsa48	AATTGCTCCAACCTGTGGTC	
Lsa49	CCAGATGGTGGATGGAGACT	LsARF4
Lsa50	CCCCACTGCTCATCAACTTT	
Lsa51	GGTTTTATCCGCCGACAGTA	LsARF5b
Lsa52	GACCGACTTCCGGTATCTCA	
Lsa53	TGTGCAGGGTGATTGATGTT	LsARF10
Lsa54	TGTTGCGTTTGTGTGTCTCA	
Lsa55	ACATATGTCCGCCTCCATTC	LsARF7a
Lsa56	CAGGGAAAATGGAACTCGAA	
Lsa57	TGATGACATTCCGTCGTGTT	LsARF19a
Lsa58	CACATCTGGCTTTTGCTGAA	
Lsa59	GCCAAGATACTTTGCCGTGT	LsARF2b

Lsa60	CTGTGATTCTGGTGGTGGTG	
Lsa61	GTGAGTGAAGAAGGGCAATG	18s rRNA
Lsa62	CACTTTCAACCCGATTCACC	
Lsa69	GACAGTTTCACAAAGTCGATTAA	LsFT
Lsa70	TGTGAAAAGCCCGGAGG	
Lsa71	CTAGAGAAAGACATACCCTCCCAC	LsAP1
Lsa72	TCACTTGTTCATGTGTTGAATCA	
Lsa73	TGCCGGAAGAGGGTGAAAA	LsAP3
Lsa74	TCAAGGAAGGCGATGATCATG	
Lsa75	TCTGTCATGCTGAACGCAGC	LsLFY
Lsa76	CTAAAACTGGAGATGACCACCACC	
Lsa77	AATCCAAACAGACCAAGGAACAG	LsSOC1
Lsa78	ACTTTGACCATCTTTGCTTCGTT	
Lsa79	agaggaggacctgcatatgATGAAGCTTTCAACATCAGGGTCTG	LsARF8a,
Lsa80	cgacggatccccgggaattcAAAATCAAGTGACCCGGACCCCAT	
Lsa81	agaggaggacctgcatatgATGAAGCTTTCAACATCAGGGTCTG	LsARF8a,
Lsa82	cgacggatccccgggaattcTTGAAAAGATGAAGCTCCAGGGTA	
Lsa83	agaggaggacctgcatatgGGAGACAGCAGAGATGATGCAGT	LsARF8a,
Lsa84	cgacggatccccgggaattcAAAATCAAGTGACCCGGACCCCAT	

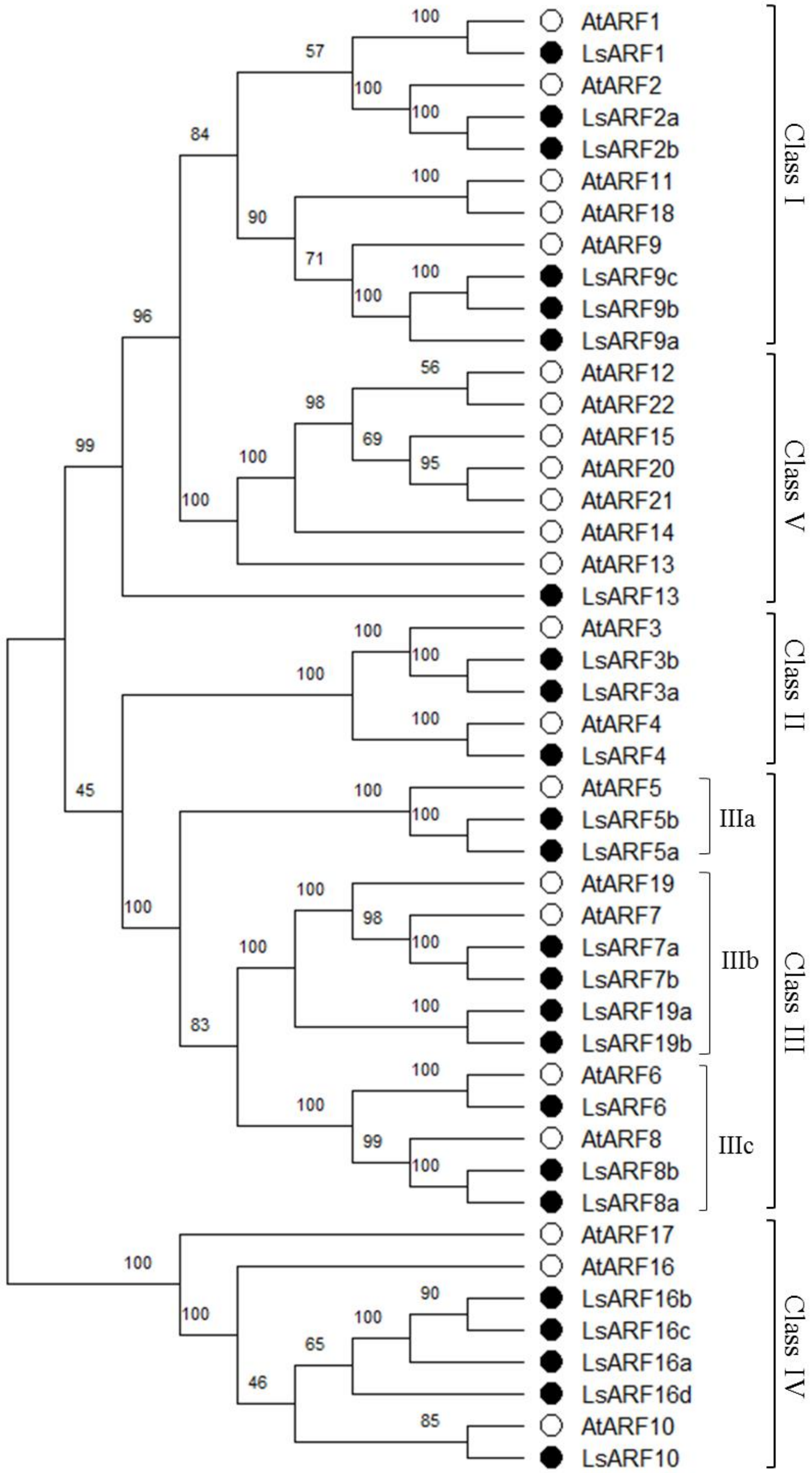

Supplementary Figure S1. Phylogenetic relationship between Arabidopsis and lettuce ARFs. Based on the ARF protein sequences of Arabidopsis and lettuce, a neighbor-joining tree was generated using MEGA X with default settings.

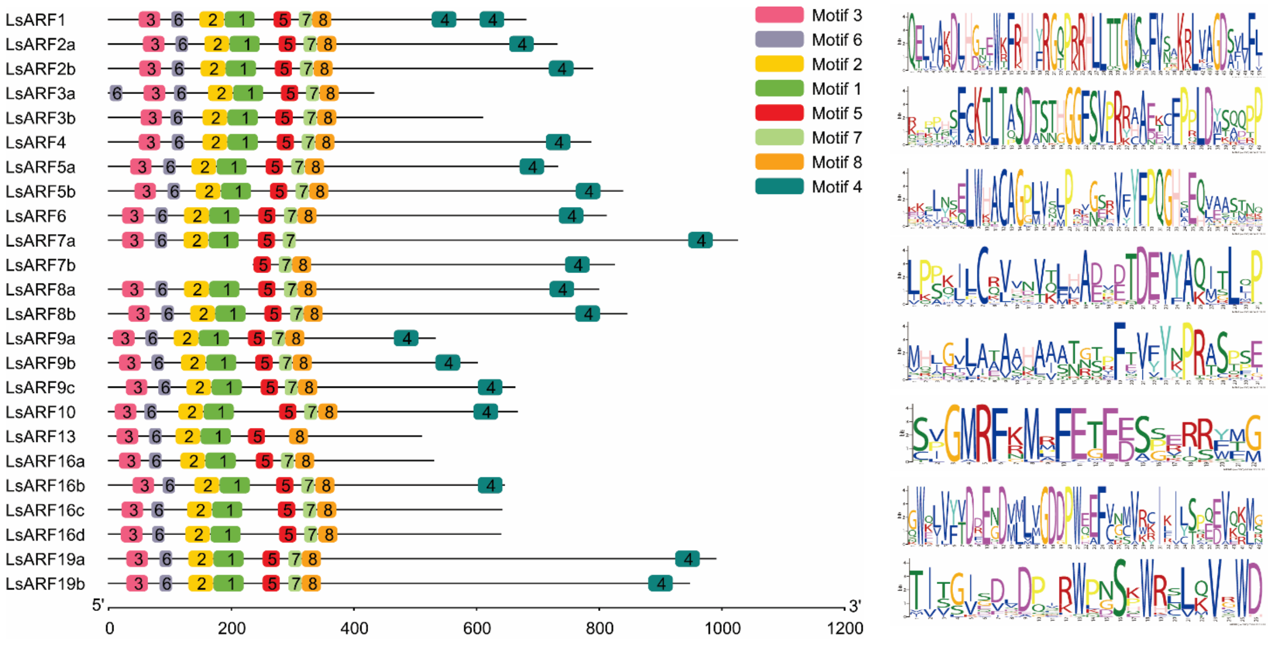

Supplementary Figure S2. The conserved motifs of LsARFs. All motifs were identified by MEME with the complete amino acid sequences of LsARFs. The rectangles in different colors on the right represent the different motifs with the consensus sequences shown on the right alongside.

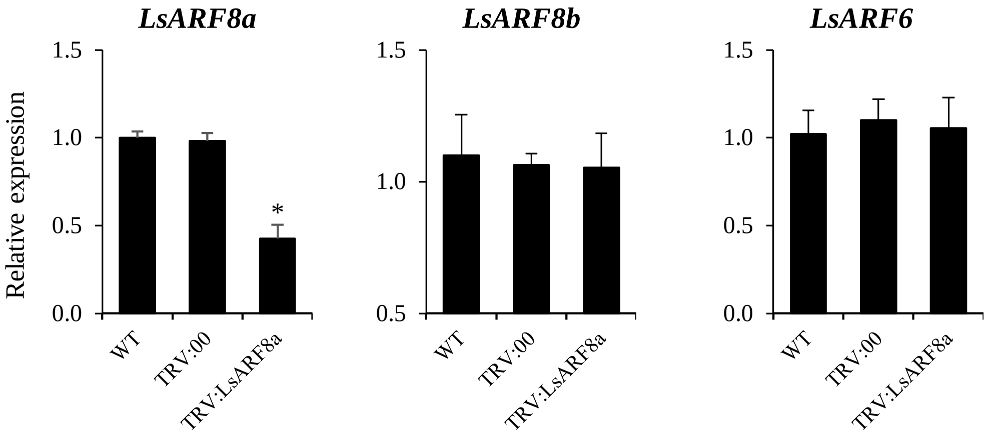

Supplementary Figure S3. **qRT-PCR analysis of *LsARF8a, LsARF8b, and LsARF6* in *LsARF8a*-silencing and control lettuce plants.** Error bars indicate SE from three biological replicates, and asterisks indicate statistically significant differences between WT and controls, as determined by the Student's *t*-test (*, p<0.05).

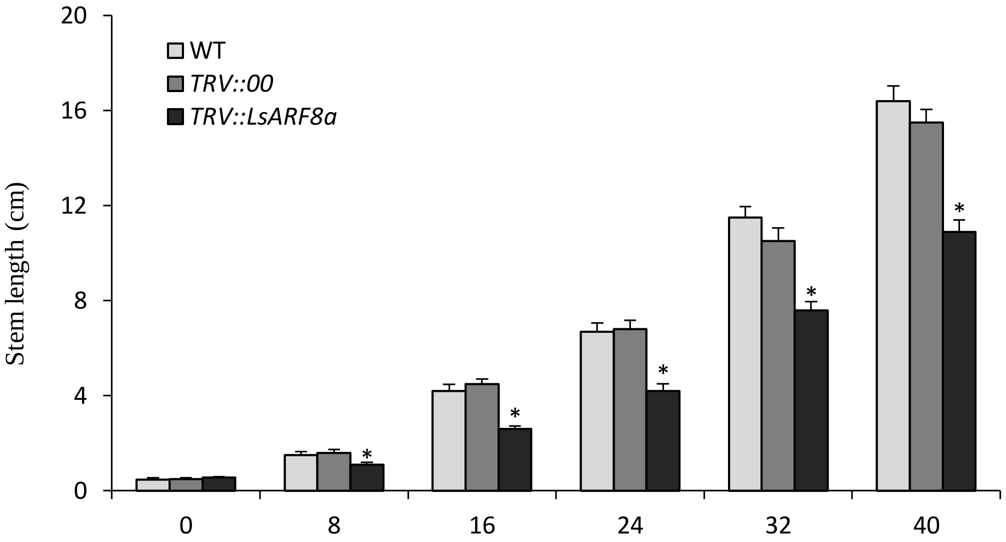

Supplementary Figure S4. Stem length changes of *LsARF8a*-silencing and control lettuce plants under warm temperatures. Error bars indicate SE of the mean (N=10), and asterisks indicate statistically significant differences between WT and treatments, as determined by the Student's *t*-test (*, p<0.01).
